# Beyond a Climate-Centric View of Plant Distribution: Edaphic Variables Add Value to Distribution Models

**DOI:** 10.1371/journal.pone.0092642

**Published:** 2014-03-21

**Authors:** Frieda Beauregard, Sylvie de Blois

**Affiliations:** 1 Department of Plant Science, McGill University, Sainte Anne-de-Bellevue, Quebec, Canada; 2 Department of Plant Science and McGill School of Environment, McGill University, Sainte Anne-de-Bellevue, Quebec, Canada; Cirad, France

## Abstract

Both climatic and edaphic conditions determine plant distribution, however many species distribution models do not include edaphic variables especially over large geographical extent. Using an exceptional database of vegetation plots (n = 4839) covering an extent of ∼55000 km^2^, we tested whether the inclusion of fine scale edaphic variables would improve model predictions of plant distribution compared to models using only climate predictors. We also tested how well these edaphic variables could predict distribution on their own, to evaluate the assumption that at large extents, distribution is governed largely by climate. We also hypothesized that the relative contribution of edaphic and climatic data would vary among species depending on their growth forms and biogeographical attributes within the study area. We modelled 128 native plant species from diverse taxa using four statistical model types and three sets of abiotic predictors: climate, edaphic, and edaphic-climate. Model predictive accuracy and variable importance were compared among these models and for species' characteristics describing growth form, range boundaries within the study area, and prevalence. For many species both the climate-only and edaphic-only models performed well, however the edaphic-climate models generally performed best. The three sets of predictors differed in the spatial information provided about habitat suitability, with climate models able to distinguish range edges, but edaphic models able to better distinguish within-range variation. Model predictive accuracy was generally lower for species without a range boundary within the study area and for common species, but these effects were buffered by including both edaphic and climatic predictors. The relative importance of edaphic and climatic variables varied with growth forms, with trees being more related to climate whereas lower growth forms were more related to edaphic conditions. Our study identifies the potential for non-climate aspects of the environment to pose a constraint to range expansion under climate change.

## Introduction

Climate is a strong predictor of plant species distribution at regional and continental scales, and therefore climate change is expected to lead to range shifts [1]. Models that predict the impact of climate change on plant distribution, however, often ignore the relative contribution of other potentially important environmental predictors that could limit plant species' ability to establish in areas newly within their climatic niches. When abiotic predictors other than climate are included in species distribution models, they are often those that can be interpreted across large expanses/grain sizes, such as the ones derived from digital elevation models, generalized geological characteristics, or satellite imagery [2]–[4]. Edaphic variables that are typically measured at point locations in the field and that vary at fine spatial scales, such as pH or humus characteristics, are rarely considered in regional or continental assessments, and so their contribution to distribution models relative to that of climate variables remains largely untested.

Commonly, the climatic signal when measured over broad climatic gradients is expected to override the influence of edaphic variables in distribution models [5], with only marginal gain to model fit obtained from adding edaphic data [6]. However, recent studies have also shown that including edaphic variables, along with climate variables, can greatly influence predicted species distribution even at large regional extents, with important consequences for predictions of range expansion or contraction under climate change [7]–[11]. As these studies have focused on a few woody species and grasses, it is recognized that this work needs to be extended to a larger suite of species, growth-forms, and regions since it cannot be assumed that all species would respond to climatic or edaphic gradients uniformly [10], [12], [13].

A common limitation in incorporating edaphic variables in distribution models is the availability of data over large spatial extents. Several edaphic variables are categorical in nature (e.g., drainage class or soil type) and cannot meaningfully be averaged within the large grid cells commonly used in distribution models. Ideally, species presence or absence must be recorded at the location where the edaphic variables are measured, which is often not the case when species are recorded in grid cells. As well, species occurrence records are often obtained from compiled sources such as the Global Biodiversity Information Facility (www.gbif.org) or herbaria which do not provide edaphic information. Consequently, even if the choice of variables in predicting species distribution should ideally be based on the known ecological requirements of species [6], [7], [10], [14], [15], so that projections are more robust for new areas or time frames [16], [17], few modelling frameworks have incorporated potentially ecologically relevant sets of predictors beyond climate variables. Regions for which information about species occurrence patterns precisely matches the location of edaphic data therefore provide invaluable model systems to assess the relative contribution of climatic and edaphic conditions to plant species distribution and can improve our knowledge of factors limiting species' ranges in a climate change context. Transitional areas between major ecosystems such as that between the northern temperate forest and the boreal forest are ideally suited to test this, since they show important gradients in both climatic and edaphic conditions.

We used an ecological dataset of global significance (one of the 20 largest datasets listed in the Global index of vegetation plot databases, www.givd.info [18]) to test a series of hypotheses about the relative importance of climatic and edaphic conditions on the distribution of plant species. Here we consider edaphic variables broadly to be those describing the nature of the site's substrate, including the topographic position. This forest database covers a large geographic area in eastern North America with a diverse climate, yet with a grain size appropriate to capture the variability of edaphic features. First, we asked whether climate-only species distribution models (cSDM) have better predictive accuracy than edaphic-only models (eSDM); we also tested whether combined edaphic-climatic models (ecSDM) had improved predictions over models with only climate predictors. Second, since growth-forms are associated and adapted to climate regimes [19], we considered whether the value of including climate vs. edaphic variables varied with growth-forms (trees, shrubs and sub-shrubs, seed bearing herbaceous plants, non-seed bearing plants and lichens). The buds of trees and shrubs are exposed to harsh climatic conditions, whereas low-lying species may benefit from more sheltered conditions and micro-climates, or their perennating buds may escape unfavourable climatic conditions underground. Finally, we verified commonly-held assumptions about the relationship between biogeographic attributes of a species within the study area and model outcomes [20], [21]. We expected prevalent species in the study area to have models with lower predictive accuracy than less prevalent species because the latter would be restricted to more specific abiotic conditions. As well, since climate has a strong latitudinal gradient in the study area, we expected it to be a better predictor for species with a range boundary in the study area than for species without a range boundary.

This study shows the potential for fine scale edaphic variables to improve species distribution models even over a large regional extent and climate gradient, while also confirming the greater importance of climate at this scale in controlling distribution. It also highlights previously unrecognized relationships between climatic and edaphic predictors and growth-forms.

## Materials and Methods

### Study area

**Figure 1:**
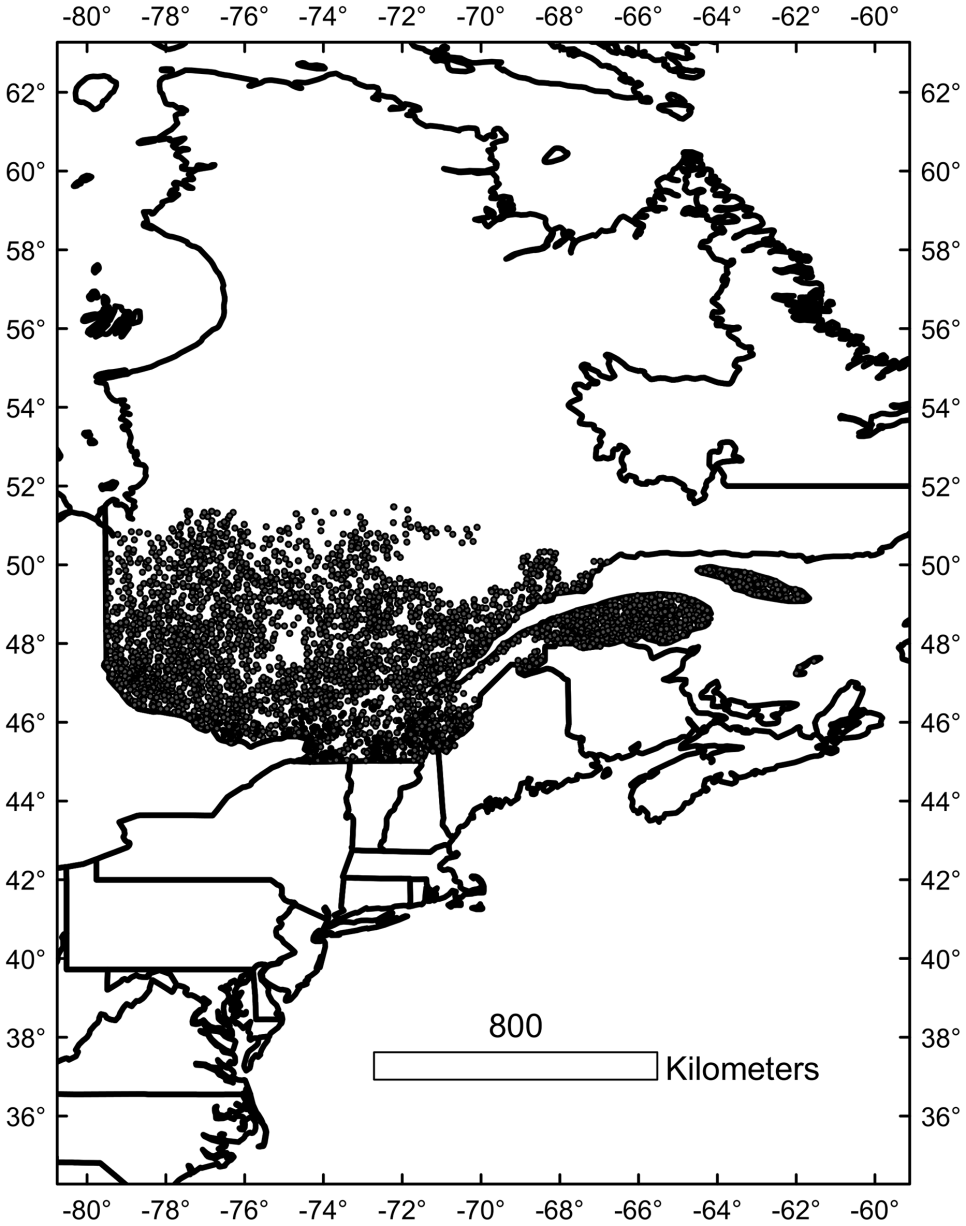
Map of study area showing sampling point locations.

The study was carried out in southern Quebec, Canada (south of 52° N, Figure 1). The study area covers more than 55 million hectares. Two major vegetation zones are present: the northern temperate zone in the south, and the boreal zone in the north, with a gradient in importance of broadleaved trees in the south to evergreen trees in the north [22], [23]. Temperature follows a north/south gradient with average annual temperatures ranging from 6.5°C in the south to −4.5°C in the north. The precipitation pattern is more longitudinal, with the western side of the province having lower annual precipitation than the east; total annual precipitation ranges from 730 mm to 1500 mm [24]. Edaphic characteristics are also diverse. The main soil types are glacial tills, clay and sandy soils from lacustrine and fluvial origins, and peat bogs and marshland with organic soils. There are several mountain ranges in the study area, which have different geologic histories and add to the diversity of soil conditions. The Canadian Shield underlies much of the study area, thus many sites have acidic bedrock. The Appalachian Mountains contain pockets of carbonate rocks (calcareous rocks, dolomites, and marbles). In the Saint Lawrence Lowlands, there are rich lacustrine deposits [22].

### Data sources

Species and edaphic data were obtained from the Quebec Ministry of Natural Resources (*Ministère des Ressources naturelles du Québec*) Vegetation Database of Quebec, the original name for this database is the *Point d*'*Observation Écologique* (POE) [25]. This dataset is available through the Ministry upon their approval of its intended use. At 4,839 uninhabited locations within the study area, 400 m^2^ circular quadrats were sampled for the presence of a set list of 314 plant species along with detailed edaphic characteristics. Some variables were obtained via photo-interpretation. The sampling was made between 1987 and 2000. A detailed description of the methods used for data collection can be found in Saucier *et al.* [26] and are briefly outlined below. Edaphic variables that we used for modelling and their units or categories are given in Tables 1 and 2. They were measured as follows: elevation (taken from a 1∶20 000 topographic map), relative height (height of the site relative to the surrounding areas taken from a 1∶250 000 topographic map, or from a 1∶15 000 or 1∶40 000 aerial photo if available, or in the field), slope position (assessed in the field), slope angle (measured with a clinometer at the centre of the plot), microtopography (assessed visually in the field with a pictogram key), drainage (assessed in the field based on natural drainage class keys following the Canadian system of soil classification), speckling (indicative of soil saturation, assessed visually within the soil profile), parent material (assessed visually in the field with keys), texture of the B horizon (using tactile tests in the field to match textural classes from the Canadian system of soil classification), humus type (assessed in the field using keys), humus depth (assessed in the field visually and by touch, up to a depth of 1 m), and soil profile depth (assessed in the field visually to the depth of the start of the BC horizon, or the C horizon if present). pH was measured in the field using a Hellige-Truog test kit for several horizon depths; we grouped and averaged these into: pH of surface horizons (all humus layers and A horizons for which pH was measured, except for the eluviated A horizon, which was not consistently taken), pH of B horizons (all B horizons measured above transitional BC horizons), and the pH of the BC or C horizon (the pH taken at the deepest level). Some potentially relevant variables in the database (e.g., depth of the water table, exposure) were not included because of a lack in consistency in their measurements or because the measure included combinations of continuous and categorical values.

**Table 1:** Continuous variables used in modelling.

Variable name	Form	Mean	SD
Humus depth (cm)	Edaphic	9.9	6.9
Soil depth (cm)	Edaphic	32	15.7
pH surface horizons	Edaphic	4.4	0.7
pH B horizons	Edaphic	5.8	0.8
pH BC or C horizon	Edaphic	6.7	0.7
Elevation (m)	Edaphic	338	151
Slope angle (%)	Edaphic	11	11.2
Degree days (accumulated first to last frost, 5°C base)	Climatic	1341	254
Minimum temperature (°C)	Climatic	−21	3.6
Precipitation (accumulated April to September, mm)	Climatic	560	58

**Table 2:** Categorical variables used in modelling.

Variable name†	Categories (count)
Relative height	Higher (1000), level (1940), lower (1898)
Humus type	Peat (310), mull (184), moder (1180), mor (3164)
Slope position	Flat (892), depression (364), plateau (848), mid-slope (2075), crest (122), upper slope (457), lower slope (80)
Microtopography	Even (1590), uneven (2140), very uneven (895)
Speckling	Colouration from good aeration (2148), colouration from continuous saturation (2148), colouration from alternating flooded and dry conditions (691)
Texture B horizon	Sand (328), sandy loam (733), loam (1325), loamy sand (1062), clay (154), clay loam (263), loamy clay (890), loamy sandy clay (83)
Parent material origin	Glacial (3015), glacio-fluvial (458), fluvial (55), marine (259), estuarine (42), laucustrine (528), colluvial (262), bed rock close to the surface (102)
Drainage	Excessively drained or somewhat excessively drained (62), well drained (1362), moderately well drained (2254), somewhat poorly drained (939), poorly drained or very poorly drained (221)

† All are edaphic variables

All the species modelled are native to the study area and include bryophytes and lichens. Out of the 314 surveyed species, 128 met our requirements of having at least 100 occurrences, and so were retained for modelling (Table S1). This minimum sample size level was chosen because species distribution model accuracy has been shown to decrease with less than 100 occurrences [27], [28].

Thirteen climate variables (Table S2) for the period 1961–1990 were obtained from the USDA Forest Service Rocky Mountain Research station website (http://forest.moscowfsl.wsu.edu). They were produced using Hutchinson's ANUSPLIN software, which uses a digital elevation model and creates thin-plate spline interpolations of weather station data [29]. These surfaces were produced for North America from data from 11,757 weather stations, 471 of these were within our study area. Full details on the methods used for creation of these surfaces are available in Rehfeldt [30]. Climate data have a resolution of 0.0083 decimal degrees (≈1 km). The sampling points of the POE data were matched to this grid, and the climate data was joined to the other environmental variables for each data point.

### Data preparation

Preliminary analysis revealed that climate variables relating to temperature were highly correlated with each other, as were indices derived from precipitation and moisture (such as precipitation-potential evapotranspiration). A pre-selection of climate variables was made to reduce this multicollinearity using the VARCLUS procedure [31] with SAS 9.2 software (SAS Institute Inc., 2008). VARCLUS is an algorithm that produces clusters of variables that have similar patterns of variation. It is an iterative process that works by splitting variables into groups based on which of the first two principal components within a group has the highest correlation, as well as by iteratively reassigning cluster membership to test the effect on this classification. We entered the 13 climatic variables (Table S2) into the clustering analysis, and selected for further analysis one variable from each cluster based on their having a high R^2^ with their own cluster, a low R^2^ with the other clusters, and on their biological and physiological significance for plants. Climate variables retained were growing degree days (base of 5°C), growing season precipitation (total from April to September), and minimum temperature of the coldest month.

Tests for multicollinearity were further done between all the selected variables (edaphic and climatic). Pearson correlation coefficients and R^2^ statistics were calculated between all of the continuous variables. For ordinal and categorical variables, contingency tables were made and the significance of a chi square test was used to assess multicollinearity. One-way ANOVAs were performed between each categorical or ordinal variable and each continuous variable. For all tests, although there were several significant relationships at a P<0.05 threshold, none had a R^2^ higher than 0.3, and so no further variables were removed. These analyses were made in Statistica 10.0 (Statsoft, Inc., 2010).

### Modelling

#### Model construction

Models were constructed for each species using either climate variables, edaphic variables, or both sets of variables. We chose our modelling approach to be comparable to commonly used SDM techniques, including both model-driven and data-driven approaches to model fitting [15]. Because the choice of a statistical model can influence the result, we tested four statistical model types: generalized additive model (GAM), generalized boosted model (GBM), generalized linear model (GLMs), and Random Forest model (RF) within the BIOMOD platform [32] implemented in R 2.12.1 (R Development Core Team, 2010). For all models a version of a step-wise or iterative approach to model fitting was used, so that the final model may not have included all provided variables. For GLM and GAM, both forward and backward selections were made. GBM and RF both work by producing many models; these are weighted, such that some variables will have greater importance in predicting the outcome than others. We used Akaike's Information Criteria to compare competing model types within this fitting process. For the GLMs, quadratic terms were allowed, but interactions between model terms were not, since this would have created many possible predictors, especially since there were already many categorical variables in the analysis. For GAMs, interaction terms were also not included and degrees of freedom for smoothing were set at three, which is comparable to a quadratic response [15]. GBM and RF include interaction terms between variables because of their tree structure. Ten iterations for each species were made with 70% of the species occurrence data used to fit the models, and 30% to test the models (referred to from now on as cross-validation models); also a full data model was made with 100% of the available data.

#### Model predictive accuracy

Model predictive accuracy was assessed with the true skills statistic (TSS) and area under the receiver operating characteristic curve (AUC). We calculated these for both the test data for the cross-validation models and for the full data model. The TSS is similar to the Kappa statistic, however, unlike Kappa, the TSS is not as sensitive to prevalence. TSS is calculated as the sensitivity plus the specificity minus one [33], [34]. The threshold chosen for ranking a point as present or absent in order to calculate the specificity and sensitivity was based on the value that would maximise the TSS score. We consider values of TSS greater than 0.6 to be good, 0.4 to 0.6 moderate, and less than 0.4 poor ([35] adapted from [36]). The AUC is taken from the receiver operating characteristic curve which is the curve of sensitivity versus 1 minus the specificity for a range of probability threshold values for ranking points as present or absent. The area under the curve (or AUC) is then used as a measure of model predictive accuracy [34]. We consider AUC values greater than 0.8 to be good, between 0.6 and 0.8 to be moderate, and less than 0.6 to be poor ([37] adapted from [38]). To compare eSDMs, cSDMs and ecSDMs we made a t-test of the ten cross-validation scores of the TSS and AUC of the competing model types for each species. We also made linear regressions between the average cross-validation scores for each predictor set. An investigation of the spatial distribution of this prediction success was also made by mapping the values from the confusion matrix [34] for the different SDMs.

#### Variable importance estimation

To evaluate the importance of variables with the ecSDMs, the full data models for all statistical model types were used and a measure of variable importance was calculated as one minus the correlation between the model output and the model output with the variable of interest permutated [32]. This metric was chosen because it is comparable across all the statistical model types. High values indicate greater importance.

### Species characteristics

We compared species based on their characteristics to test if there were general patterns in model predictive accuracy and variable importance. The characteristics considered were: (1) the presence of a range boundary within the study zone; species ubiquitous in the study area were given a status of no range boundary. Species with no observations in the north, south, east or west of the study site (or combinations) were given the status of having a range boundary. In order to determine this, histograms of species presence with latitude and longitude were made to identify clear breaks in species prevalence, so that species that had no observations within regions of the study zone could be identified and distinguished from species that were merely rare on the landscape. In case any species had a more complex distribution that was not a north-south or east-west split, maps of each species were also examined. All of the categorisation was done prior to analysis/modelling (results not shown); (2) species prevalence in the sampled sites (a count of occurrences), and (3) plant growth forms of trees, shrubs and sub-shrubs, herbaceous seed bearing plants, or herbaceous non-seed bearing plants and lichens. The significance of these characteristics on model predictive accuracy and variable importance were tested with either one-way ANOVAs, t-tests (Welch's), or Pearson's correlations where appropriate, as well as counts of the number of species in each group with at least one important edaphic predictor.

## Results

### Predictive accuracies across models

When comparing all models, many had TSS and AUC values in the good range (Table 3). The ecSDM had consistently better predictive accuracy than the cSDM or eSDM for the majority of species regardless of the statistical models used. Considering both the full models and the cross-validation scores, the different statistical model types performed similarly. Of note, RF was the most sensitive to data input/over-fitting, producing full models with perfect classification of presence and absence points but having cross-validation scores that were much lower than these full model scores. Despite this, the RF models can be considered as robust as the other statistical model types, since their cross-validation scores were comparable. There was a large difference when using the TSS or AUC as an assessment of model accuracy, although the two were highly correlated. Based on the threshold levels chosen, the TSS metric produced a more conservative estimate of the number of good models than the AUC metric. Because there was no great difference between statistical models, the rest of the results are presented for the average of the four models.

**Table 3:** Summary statistics of predictive accuracy for the different models.

	Full model		Cross validation models	
**Climate SDM**	AUC	TSS	AUC	TSS
*GAM*	0.79±0.09 (50%)	0.48±0.17 (27%)	0.78±0.10 (50%)	0.48±0.18 (23%)
*GBM*	0.85±0.07 (75%)	0.56±0.15 (41%)	0.80±0.09 (55%)	0.50±0.16 (27%)
*GLM*	0.79±0.10 (49%)	0.48±0.12 (23%)	0.78±0.10 (50%)	0.45±0.12 (24%)
*RF*	1±0 (100%)	0.97±0.02 (100%)	0.78±0.09 (50%)	0.46±0.17 (22%)
**Edaphic SDM**				
*GAM*	0.81±0.07 (56%)	0.49±0.12 (22%)	0.78±0.07 (43%)	0.45±0.12 (13%)
*GBM*	0.81±0.06 (62%)	0.49±0.11 (18%)	0.77±0.07 (34%)	0.43±0.12 (8%)
*GLM*	0.81±0.07 (56%)	0.48±0.12 (23%)	0.78±0.07 (42%)	0.45±0.12 (12%)
*RF*	1±0 (100%)	1±0 (100%)	0.78±0.07 (43%)	0.44±0.12 (12%)
**Edaphic-climate SDM**				
*GAM*	0.85±0.07 (79%)	0.57±0.15 (43%)	0.83±0.08 (62%)	0.54±0.15 (37%)
*GBM*	0.86±0.07 (83%)	0.59±0.13 (44%)	0.83±0.07 (63%)	0.53±0.14 (34%)
*GLM*	0.85±0.07 (76%)	0.57±0.15 (41%)	0.82±0.08 (63%)	0.53±0.15 (36%)
*RF*	1±0 (100%)	1±0 (100%)	0.84±0.07 (71%)	0.55±0.14 (38%)

*TSS*-true skill statistic and *AUC*-the area under the receiver operating characteristic curve; *climate SDM*-species-distribution model using only climate variables; *edaphic SDM*-species distribution model using only edaphic predictors; *edaphic-climate SDM*-species distribution model using both edaphic and climate predictors. Reported are means for all species for each statistical model type, ± one standard deviation, and percentage of species with a AUC greater than 0.80 or a TSS greater than 0.60; for cross validation models, the mean of each species was first calculated

**Figure 2:**
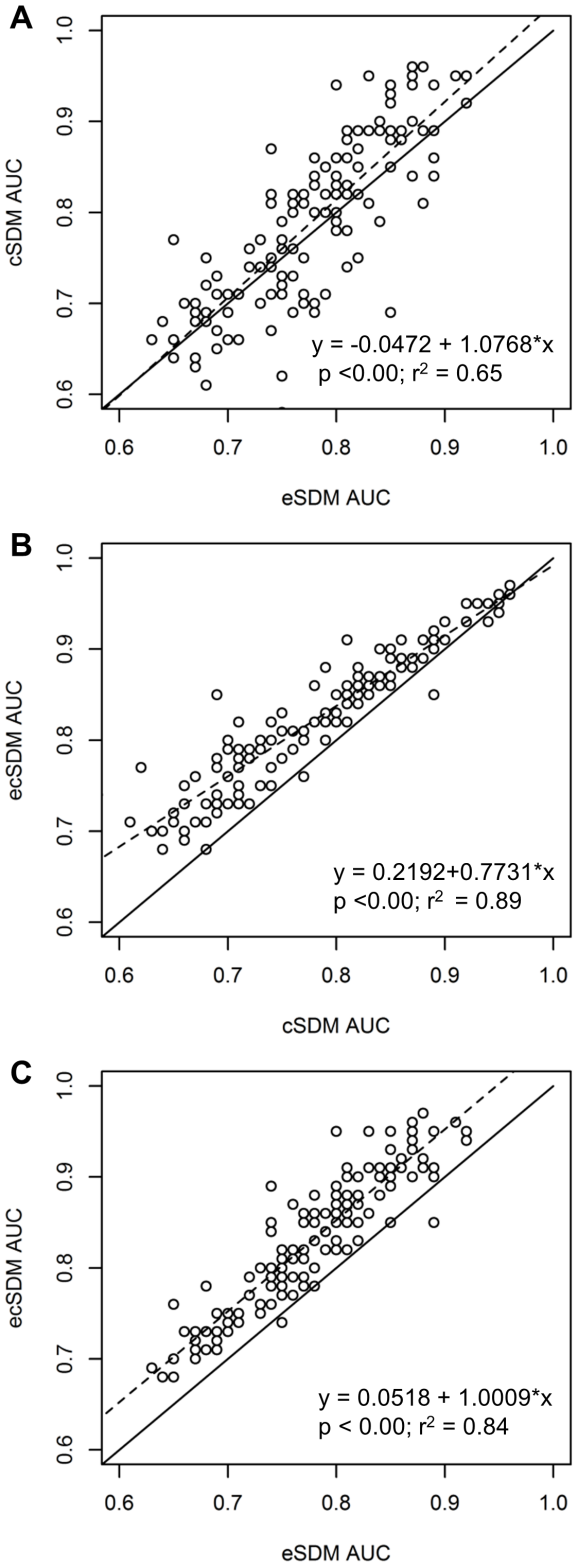
Comparisons of predictive accuracy of different abiotic model types through scatterplots and regressions. Average area under the curve of the receiver operator characteristic (AUC) for each species (mean of the 10 x cross-validation models of each statistical model type); solid lines have a slope of one with no intercept; dashed lines are the linear regression produced from either A) the AUC of the climate species distribution models (SDM) vs. AUC of the edaphic SDM; B) the AUC of the edaphic-climate SDM vs. the AUC of the climate SDM; or C) the AUC of the edaphic-climate SDM vs. the AUC of the edaphic SDM. Panel A: there is a small improvement to model fit if models are constructed from climate vs. edaphic predictors; panel B: improvement is greatest for models with lower predictive accuracy; panel C: improvement is consistent across strong and weak models, and is greater than that from adding edaphic predictors to climate models.

The improvement to model predictive accuracy obtained by including the other set of abiotic predictors (Table 3, Figure 2) was greater for the eSDMs, and more species had models with higher predictive accuracy if only including climate variables than if only including edaphic variables. The pattern of improvement to model predictive accuracy is illustrated in Figure 2 for the AUC metric, the TSS metric showed similar patterns (but with lower values which are characteristic of the TSS metric).

Model predictive accuracy was generally higher for species with a range boundary within the study zone, but was not significant for the ecSDM (Table S3). As well, less prevalent species had cSDM and eSDM with higher predictive accuracy, reflected in the significant negative correlation between model predictive accuracy and prevalence, but there was no effect on the full ecSDMs (Table S3). There was a clear difference, however, in the general spatial patterns of omission and commission errors for species with a range boundary. The spatial distribution of the predictive success based on the confusion matrix is illustrated in Figures S1–S19 for those species with a north-south range boundary in the study area and an average AUC for all three sets of environmental predictor models of at least 0.90 (N = 19). The cSDMs predicted most sites as positive within the area where a species was prevalent, but few sites outside this area as positive. In contrast, the eSDM predicted sites as positive both within and outside the areas where the species was most prevalent.

We also considered the effect of plant growth form on the predictive accuracy within each set of abiotic predictors. For the ecSDM, ANOVAs of plant growth form and model predictive accuracy had significant differences (P<0.05) for both the TSS and AUC metrics, with trees having a higher mean (TSS  = 0.69, AUC  = 0.89), than shrubs (TSS = 0.62, AUC = 0.86), herbaceous seed bearing species (TSS = 0.59, AUC = 0.84) and non-seed bearing species and lichens (TSS = 0.58, AUC = 0.84). Tukey's honest significant difference test identified the ecSDM for trees as having a significantly higher predictive accuracy than those for herbaceous seed bearing species. Growth form did not have significant differences on cSDM and eSDM predictive accuracy based on ANOVAs.

To highlight species which had the greatest improvement by adding the edaphic data, we considered those species which had an improvement in model predictive accuracy (AUC scores) in the 90^th^ percentile when comparing the cSDM to the ecSDM (Table 4). There was no discernible pattern to the characteristics of these species. They came from all the plant growth form types; they included species with and without range boundaries, and varied in terms of their prevalence in the study area. Of note is that three of the four *Sphagnum* species were in this group.

**Table 4:** Species with the greatest improvement to model predictive accuracy (top 90^th^ percentile) when adding edaphic predictors to climate only models.

Scientific name	Edge	Count	cSDM AUC	ecSDM AUC
*Actaea rubra* (Aiton) Willd.	+	1194	0.74	0.82
*Alnus incana* (L.) Moench. ssp. rugosa (Du Roi) Clausen	-	905	0.71	0.82
*Athyrium filix-femina* (L.) Roth	+	784	0.69	0.77
*Coptis trifolia* (L.) Salisb.	-	1804	0.61	0.71
*Epigaea repens* L.	+	213	0.70	0.79
*Ilex mucronata* (L.) Powell, Savolainen & Andrews	-	983	0.71	0.79
*Larix laricina* (Du Roi) Koch	-	222	0.62	0.77
*Lycopodium obscurum* L.	+	1565	0.67	0.76
*Maianthemum trifolium* (L.) Sloboda	+	222	0.81	0.91
*Mitella nuda* L.	+	198	0.79	0.88
*Populus grandidentata* Michx.	+	170	0.75	0.83
*Prunus pensylvanica* L.	+	1347	0.78	0.86
*Rubus pubescens* Ruf.	+	1248	0.69	0.78
*Sphagnum girgensohnii* Russow	+	580	0.70	0.80
*Sphagnum magellanicum* Brid.	+	221	0.69	0.85
*Sphagnum squarrosum* Crome	+	123	0.58	0.74
*Thalictrum pubescens* Pursh	+	116	0.66	0.75

*Edge*-whether or not there was a range edge within the study area; *Count-*The number of occurrence points within the dataset; *cSDM AUC-*The area under the curve of the receiver operator characteristic for the climate species distribution model; *ecSDM AUC-* The area under the curve of the receiver operator characteristic for the edaphic-climate species distribution model

### Variable importance within ecSDM

If considering the mean importance of predictor variables in the full models of the ecSDM, degree days and minimum temperature were by far the most important variables (Table 5). There were a few species which had high importance of specific edaphic variables (or precipitation). To estimate objectively the number of times a variable had a “high” importance, we considered the distribution of variable importance across all species and all variables, and we found it to fit closely a negative exponential distribution, with 85% of variable importance values being below 0.1, and 95% of variable importance values being below 0.3. Based on the criteria of mean importance, count of importance between 0.1 and 0.3, count of variable importance greater than 0.3 and count of times a variable was one of the top two most predictive variables within the model, a clear pattern emerges (Table 5). Degree days is most important, with minimum temperature a close second and precipitation often important but less so than the temperature variables. Drainage, texture, humus type, humus depth, and surface pH are important for several species. Soil depth, pH of B horizons, pH of BC or C horizon, slope angle, relative height, micropotography, parent material origin and elevation are important for a few species. Slope position and speckling are never important.

**Table 5:** Variable importance across all species for the full edaphic-climatic model.

	Importance	Count >0.3	Count 0.1–0.3	Count top two variables
	(mean ± SD)			
Degree days	0.32±0.25	60	35	89
Minimum temperature	0.21±0.15	28	67	71
Precipitation	0.07±0.07	3	21	17
Humus type	0.06±0.07	2	20	16
pH surface	0.05±0.09	4	16	15
Humus depth	0.05±0.07	2	16	13
Drainage	0.04±0.07	2	17	7
Texture	0.04±0.05	0	14	9
Elevation	0.04±0.05	0	14	4
Parent material origins	0.04±0.05	1	8	5
Slope angle	0.03±0.04	0	7	0
Microtopography	0.02±0.05	1	6	3
Relative height	0.02±0.03	0	2	3
pH BC or C horizons	0.02±0.03	1	0	2
pH B horizons	0.02±0.03	0	2	1
Soil depth	0.01±0.02	0	2	1
Slope position	0.01±0.01	0	0	0
Speckling	0.01±0.01	0	0	0

*Importance*-variable importance calculated as one minus the correlation between the model output and the model output with the variable of interest randomized, for the means of the final climate-edaphic model for each statistical model; *Count-* the count of the number of species with a variable importance value greater than 0.3, which relates to the top 95% of variables importance values, or with a variable importance value between 0.1 and 0.3, which relates to the 85% to 95% margin of variable importance values; *Count top two variables-*count of the number of species with the variable among the top two most important in the model.

When considering species growth form effect on variable importance, out of the 18 variables, only soil texture, soil depth and degree days had significant differences based on ANOVAs (with P<0.05, see Table S4). Trees had a marked greater importance of degree days compared to the other groups. Although it was not usually a variable with high importance, soil depth importance was significantly higher for herbaceous plants than trees or shrubs. In least significance difference tests, but not in Tukey's honest significant difference test, soil texture was significantly more important for herbaceous plants and shrubs than for trees.

Out of the 128 species, 125 had models with at least one climate variable which had a high importance (based on a variable importance of 0.1 or greater); whereas 82 species had models with at least one edaphic variable of high importance. Considering growth form, trees had the fewest species with at least one edaphic predictor with a high importance (12 out of 30 species) and herbaceous seed bearing plants had the most (27 out of 33 species). Shrubs and seedless plants and lichens had about two thirds of species with at least one edaphic predictor having a high variable importance (21 out of 33 and 22 out of 32, respectively).

Variable importance based on t-tests (with P<0.05) was not different between the group of species with a range boundary and those without, except for degree days, which was a more important variable for species with a range boundary (means of 0.34 and 0.26, respectively), and humus depth, which was less important (means of 0.04 and 0.08, respectively). Prevalence, if significant (based on a Pearson correlation with P<0.05) was generally negatively correlated with variable importance, except for humus depth, which had a significant positive correlation (R = 0.19, P<0.05).

## Discussion

For the majority of species, temperature variables are most predictive of distribution over large geographic extents, even when grain size is suitable to capture the variation in edaphic variables. However, for some species, edaphic variables can be important predictors as well, even more so than climate predictors. Surprisingly, models made with only edaphic predictors performed almost as well as those with only climate predictors, which underlines the potential for edaphic variables to provide useful information about species distribution, even over large extents. Whereas cSDMs are definitely valuable on their own when projecting species distribution in future climate, eSDMs, or even better ecSDMs, provide useful information to help reduce the level of uncertainty of cSDMs projected into areas outside of the normal range of edaphic conditions used to train the model.

Ignoring edaphic characteristics could lead to significant overestimates of suitable conditions within a given climate or when projecting over new geographic areas (e.g., from temperate forest to boreal forest) where edaphic conditions are not equivalent to those found within the species current range. This is illustrated by the spatial distribution of errors which, despite similar predictive accuracies, was very different for the eSDMs and cSDMs (Figures S1–S19). We interpret the error pattern of the cSDM as the model being able to pick up the climatic constraints on distribution, but making many false positive predictions within the climatically suitable area. Projecting beyond the current range would lead to the same overestimate of suitable conditions. The eSDMs were able to estimate locations with suitable edaphic conditions; these fell both inside and outside the range boundaries, although with more frequency within the range boundaries. This meant that within the range boundaries, where the climate was also suitable, the model was able to accurately assign presence or absence. Outside the range boundaries, the eSDM also predicted species presences where there were suitable edaphic conditions outside of the current range/climate niche. The information garnered from the false positive locations of eSDMs could be useful to identify edaphic homologs to southern areas beyond the current range, for instance to assist migration of southern species in climate change adaptation strategies in conservation or forestry [38]. Overall, the ecSDMs were most accurate in their predictions, with many models fitting the observed distribution closely, underscoring that both edaphic and climatic aspects of the environment are important at this scale in determining species distribution.

Although all growth forms had some species with high importance of edaphic variables, distinct patterns, which are rarely emphasized, also emerged from our analysis. The distribution of trees is more constrained by climate than the distribution of other low growth forms; the latter is more related to edaphic conditions. At the landscape scale (approximately 600 ha in this case), herbaceous and shrub species were also found to be more constrained by edaphic conditions than trees [39]. Whether this means that low growth forms can escape changing climate conditions better than trees is uncertain, but their range expansion in response to climate change is expected to be more restricted by the availability of suitable edaphic conditions. On the other hand, trees, assuming they have longer life cycles than those of lower growth forms, may be more restricted in their capacity to adapt in terms of range expansion as climate warms [40]. The interactions between life history, dispersal strategy, and edaphic requirements, especially at the establishment phase, warrant further investigation in the context of climate change.

Species in the genus *Sphagnum* stood out as being better modelled if edaphic characteristics were included, drainage having a particularly high variable importance score. However, humus characteristics were also important, which brings up the issue of cause and effect between plants and their substrate. *Sphagnum* are bog species, and so are found in areas with poor drainage and thick humus layers, however, they are also the main producers of peat in the bogs in which they grow. In other words, the physical characteristics of humus may limit or promote species presence, and/or the species presence may alter humus characteristics. These complex feedback relationships may not be a problem when relating humus type or depth to the contemporary distribution of *Sphagnum*, but they would need to be carefully considered when projecting future distribution of suitable conditions in time and space. There are many other examples of the interconnected nature of vegetation and substrates. For example in our study region, tree species which grow on nutrient rich soils tend to have leaves which decompose easily, thus enhancing nutrient cycling and nutrient richness [41]. These feed-back loops are numerous and complex in ecosystems, and also not fully understood. The difficulty in pulling these relationships apart adds to the uncertainty of using SDMs to make predictions about range shifts under novel climates or geographic areas. As well, climate has an influence on soil biota and chemistry [42]. It may be best to think of these model outputs as being able to highlight areas where environmental conditions would be most favourable to a species given what is known of the current edaphic state of the landscape, but that this state will be dynamic as well, at least for some edaphic variables.

Edaphic conditions may be indicative of other physical or historical processes at the stand level [39]. Within the edaphic variables, the characteristics of the humus layer were the most predictive. The humus could be an indirect measure of several other site conditions; humus characteristics are very indicative of a site's geochemistry and the organisms it supports [43], [44]. The depth of the humus layer, for instance, is often related to disturbance regime or stand age and is expected to be greater for sites that have not been burned recently. It can also be a proxy for nutrient availability, as would humus type. Slow nutrient cycling will result in humus accumulation and these conditions could limit which species would occupy a site, thereby reducing competition. In our study, humus depth was an important variable mostly for common understory species in the boreal forest, which could explain why it was also positively correlated with prevalence.

A potential bias in favour of edaphic variables exists in our study design since we compared fifteen edaphic variables to only three climate variables, thereby increasing the chances that one of the edaphic variables would be picked up in the eSDM and ecSDMs. Whereas many climate variables are available, they are generally all derived from temperature and precipitation and therefore likely to be correlated to each other. There was, however, no justification to exclude *a priori* edaphic variables based on our preliminary analysis for collinearity. Each was fairly unique in the type of information it provided about the site, and all were plausible explanatory variables. Even with the possibility for bias towards edaphic variables, our results support climate variables as being universally important, whereas which edaphic variable relates most to species distribution tended to vary with species. We were nevertheless able to identify a few edaphic variables with consistently low contribution in this region (e.g., slope position, speckling) and future modelling could benefit from this knowledge.

We have used ‘edaphic’ variables in a broad sense to encompass all aspects of a site's physical nature, i.e. those variables relating to topography and the soil substrate and which are most often considered in ecological studies. We could have divided our predictors into further categories, for instance based on their direct or indirect effects, although these may be hard to evaluate in absolute term. Slope angle, for instance, will determine the amount of insolation, windiness, or erosion patterns and therefore probably also integrates a range of climatic and non-climatic conditions. We have not measured microclimatic conditions in this study given the grain size we used for climatic data and so some of these conditions are probably captured in some of the edaphic variables. Another possibly relevant classification could have been to distinguish between permanent site conditions (e.g., parent material, elevation) vs. dynamic ones (e.g., surface pH, humus type), assuming the latter will change with species and climate, and therefore could be less relevant when projecting in a future climate. Our results, however, suggest that both types of variables determine species distribution. More importantly, given the rapid rate of climate change, species will have to migrate and establish under current edaphic conditions.

Many SDMs are built within specific geo-political boundaries, often with no consideration for species' range boundaries [45], [46]. Our results support that model predictive accuracy is usually reduced if range boundaries are not included [20] (or if the species is common), but we observed that these effects were buffered if edaphic as well as climate variables were included when model predictive accuracy was assessed with the TSS, but not the AUC metric. As well, the importance of variables could be underestimated if an inadequate study zone is used, such as the lower importance of degree days for species without a range boundary observed in this study.

## Conclusion

In this study, we have shown that for a large suite of species native to this area, climate variables are most important in predicting distribution at regional scale, particularly for trees. Despite this, eSDMs produced models almost equal to cSDMs in predictive performance, indicating that edaphic variables also pose important constraints on distribution patterns. The inclusion of edaphic variables in SDMs significantly improved model accuracy for the majority of species, whereas the relative importance of edaphic and climatic variables varied with growth forms. In northern ecosystems such as this one, many species reach their northern edge of distribution and northern range expansion under a future warmer climate is expected [47], [48]. Our study identifies the potential for non-climate aspects of the environment, particularly variables relating to characteristics of the humus layer, to pose a constraint to this expansion. Although some edaphic characteristics are also dynamic and both species and climate are expected to modify the substrate over time, these changes are expected to happen at a slower rate than those predicted by climate models [42] and species will have to migrate under current edaphic conditions. This could result in a decoupling between edaphic and climate conditions. Edaphic SDMs could be valuable tools to locate sites edaphically-analogous to a species' current habitat in areas that are expected to become suitable under rapid climate change. This could aid in the identification of suitable refuges for conservation and management, especially for edaphically sensitive species.

## Supporting Information

Figure S1 ***Acer saccharum*** **Marsh. mapped distributions.** Comparison of omission and commission errors in the different forms of species distribution models (SDM). The statistical model used in these maps is the full data generalized linear model.
(PDF)

Figure S2 ***Betula populifolia*** **Marsh. mapped distributions.** Comparison of omission and commission errors in the different forms of species distribution models (SDM). The statistical model used in these maps is the full data generalized linear model.
(PDF)

Figure S3 ***Chimaphila umbellata*** **(L.) Bartram ssp.** ***umbellata*** **mapped distributions.** Comparison of omission and commission errors in the different forms of species distribution models (SDM). The statistical model used in these maps is the full data generalized linear model.
(PDF)

Figure S4 ***Fagus grandifolia*** **Ehrh. mapped distributions.** Comparison of omission and commission errors in the different forms of species distribution models (SDM). The statistical model used in these maps is the full data generalized linear model.
(PDF)

Figure S5 ***Fraxinus americana*** **L. mapped distributions.** Comparison of omission and commission errors in the different forms of species distribution models (SDM). The statistical model used in these maps is the full data generalized linear model.
(PDF)

Figure S6 ***Mitchella repens*** **L. mapped distributions.** Comparison of omission and commission errors in the different forms of species distribution models (SDM). The statistical model used in these maps is the full data generalized linear model.
(PDF)

Figure S7 ***Onoclea sensibilis*** **L. mapped distributions.** Comparison of omission and commission errors in the different forms of species distribution models (SDM). The statistical model used in these maps is the full data generalized linear model.
(PDF)

Figure S8 ***Ostrya virginiana*** **(Mill.) Koch mapped distributions.** Comparison of omission and commission errors in the different forms of species distribution models (SDM). The statistical model used in these maps is the full data generalized linear model.
(PDF)

Figure S9 ***Polypodium virginianum*** **L. mapped distributions.** Comparison of omission and commission errors in the different forms of species distribution models (SDM). The statistical model used in these maps is the full data generalized linear model.
(PDF)

Figure S10 ***Populus balsamifera*** **L. mapped distributions**. Comparison of omission and commission errors in the different forms of species distribution models (SDM). The statistical model used in these maps is the full data generalized linear model.
(PDF)

Figure S11 ***Prunus serotina*** **Ehrh. mapped distributions.** Comparison of omission and commission errors in the different forms of species distribution models (SDM). The statistical model used in these maps is the full data generalized linear model.
(PDF)

Figure S12 ***Quercus rubra L. var. ambigua*** **(Gray) Fernald mapped distributions.** Comparison of omission and commission errors in the different forms of species distribution models (SDM). The statistical model used in these maps is the full data generalized linear model.
(PDF)

Figure S13 ***Solidago rugosa*** **Mill. mapped distributions.** Comparison of omission and commission errors in the different forms of species distribution models (SDM). The statistical model used in these maps is the full data generalized linear model.
(PDF)

Figure S14 ***Spiraea alba*** **du Roi mapped distributions.** Comparison of omission and commission errors in the different forms of species distribution models (SDM). The statistical model used in these maps is the full data generalized linear model.
(PDF)

Figure S15 ***Tiarella cordifolia*** **L.** **mapped distribution.** Comparison of omission and commission errors in the different forms of species distribution models (SDM). The statistical model used in these maps is the full data generalized linear model.
(PDF)

Figure S16 ***Tilia americana*** **L**. **mapped distributions.** Comparison of omission and commission errors in the different forms of species distribution models (SDM). The statistical model used in these maps is the full data generalized linear model.
(PDF)

Figure S17 ***Tsuga canadensis*** **(L.) Carriere mapped distributions.** Comparison of omission and commission errors in the different forms of species distribution models (SDM). The statistical model used in these maps is the full data generalized linear model.
(PDF)

Figure S18 ***Ulmus americana*** **L. mapped distributions.** Comparison of omission and commission errors in the different forms of species distribution models (SDM). The statistical model used in these maps is the full data generalized linear model.
(PDF)

Figure S19 ***Viburnum lantanoides*** **Michx. mapped distributions.** Comparison of omission and commission errors in the different forms of species distribution models (SDM). The statistical model used in these maps is the full data generalized linear model.
(PDF)

Table S1 **List of modelled species, their characteristics and model predictive accuracy.** *Cross-validation scores*-are the means of four different statistical model types: generalized boosted models, generalized linear regression models, generalized additive models, and random forest models, each with ten iterations of data-splitting for model building and evaluation.; *SDM-* species distribution model; *AUC*-the area under the curve of the receiver operating characteristic; *TSS*-true skill statistic; *Edge*- indicates if the species had an observable range boundary within the study points; *Count-*the count of the number of occurrences.
(PDF)

Table S2 **List of climate variables included in the VARCLUS analysis.**
(PDF)

Table S3 **Model predictive accuracy and the influence of species biogeographic characteristics on these metrics.** *AUC*-the area under the curve of the receiver operating characteristic; *TSS*-true skill statistic; *SDM*-species distribution model. Reported are the means of the final climate-edaphic model for each statistical model type; comparisons of the presence of a range boundary in the study area (t-test) and the effect of number of occurrences (Pearson correlation) significant results are in bold, *P<0.1; **P<0.05.
(PDF)

Table S4 **Differing importance of variables between plant growth form groups.** Reported are significant ANOVA results of variable importance (calculated as one minus the correlation between the model output and the model output with the variable of interest randomized) for the means of the final climate-edaphic model for each statistical model type between the plant form groups: tree, shrub, herbaceous seed bearing, and seedless plants (including lichens).
(PDF)
