# Supplementary material for: Beyond a Climate-Centric View of Plant Distribution: Edaphic Variables Add Value to Distribution Models
Source: PLoS One. 2014 Mar 21;9(3):e92642. doi: 10.1371/journal.pone.0092642 (PMC3962442; doi:10.1371/journal.pone.0092642)
Supplement: Figure S5 — Fraxinus americana L. mapped distributions. Comparison of omission and commission errors in the different forms of species distribution models (SDM). The statistical model used in these maps is the full data generalized linear model. (PDF) [file pone.0092642.s005.pdf]

*Fraxinus americana* L.

False negative   True negative or absence   True positive or presence   False positive

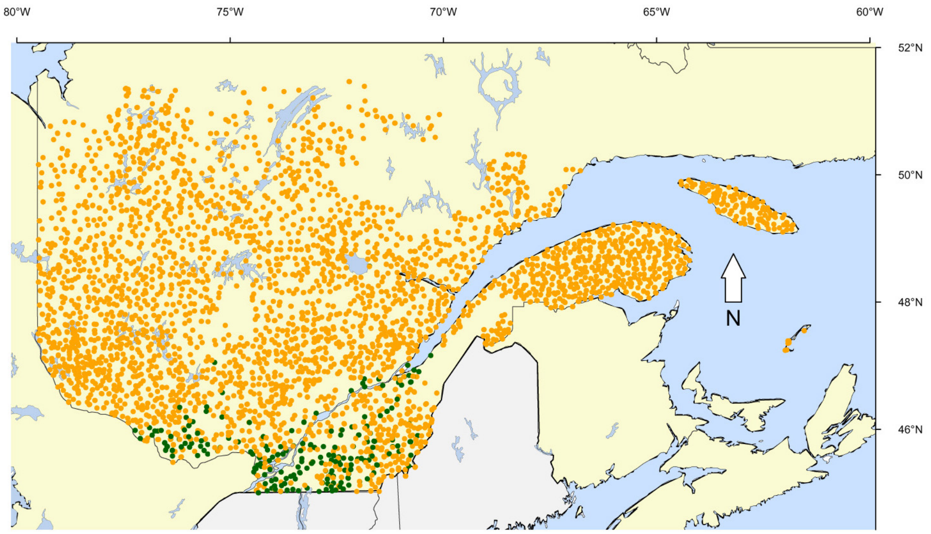


Species occurrence

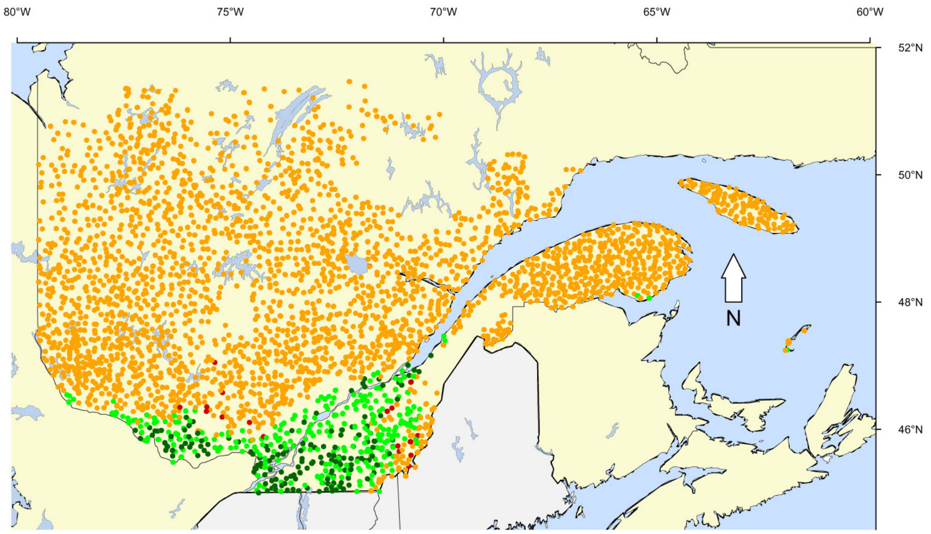


Climatic variables only
Sensitivity: 88.2% - Specificity: 88.2%

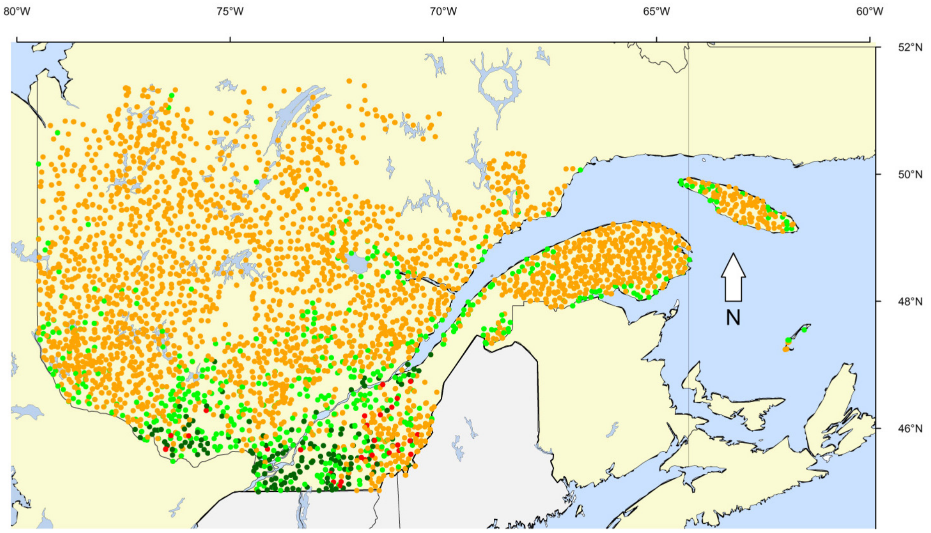


Edaphic variables only
Sensitivity: 86.1% - Specificity: 86.3%

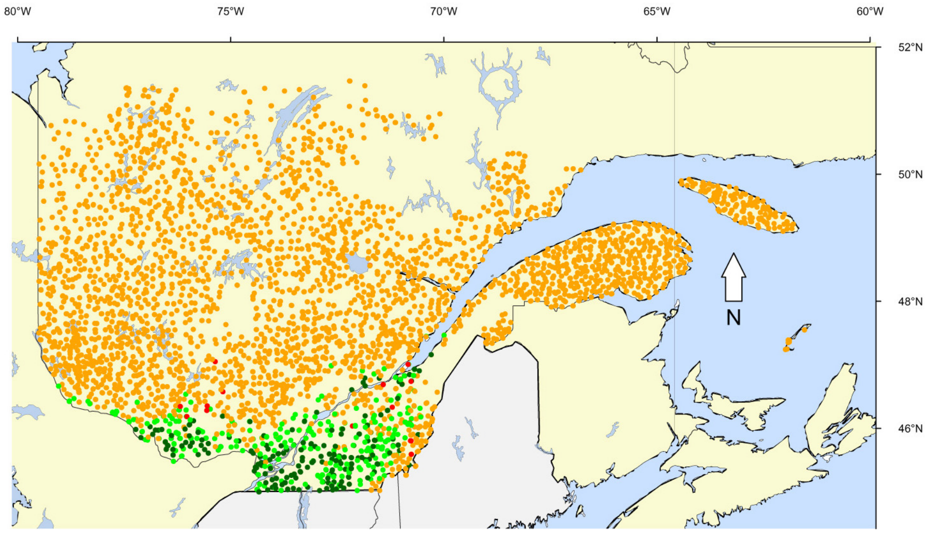


Climatic and edaphic variables
Sensitivity: 90.3% - Specificity: 90.1%
