# Supplementary material for: Beyond a Climate-Centric View of Plant Distribution: Edaphic Variables Add Value to Distribution Models
Source: PLoS One. 2014 Mar 21;9(3):e92642. doi: 10.1371/journal.pone.0092642 (PMC3962442; doi:10.1371/journal.pone.0092642)
Supplement: Figure S11 — Prunus serotina Ehrh. mapped distributions. Comparison of omission and commission errors in the different forms of species distribution models (SDM). The statistical model used in these maps is the full data generalized linear model. (PDF) [file pone.0092642.s011.pdf]

Prunus serotina Ehrh.

False negative · True negative or absence · True positive or presence · False positive

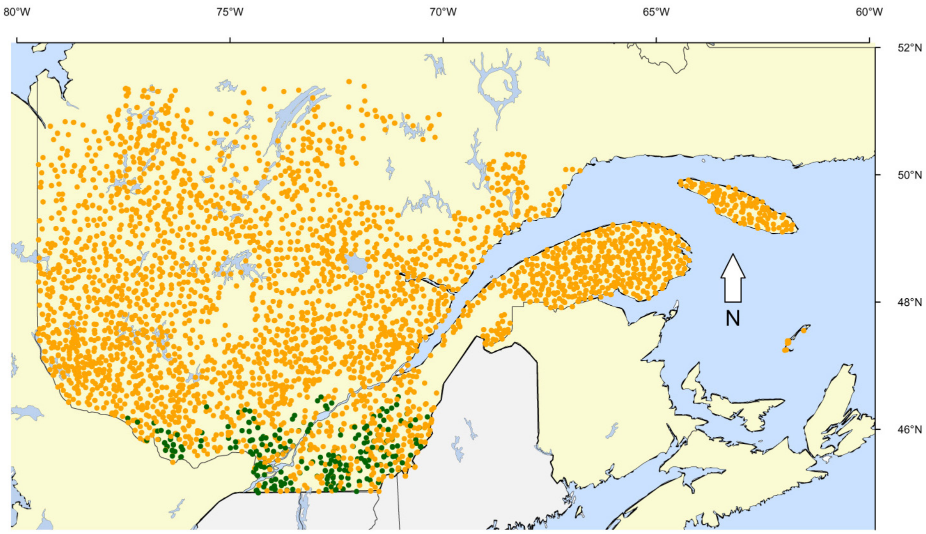

Species occurrence

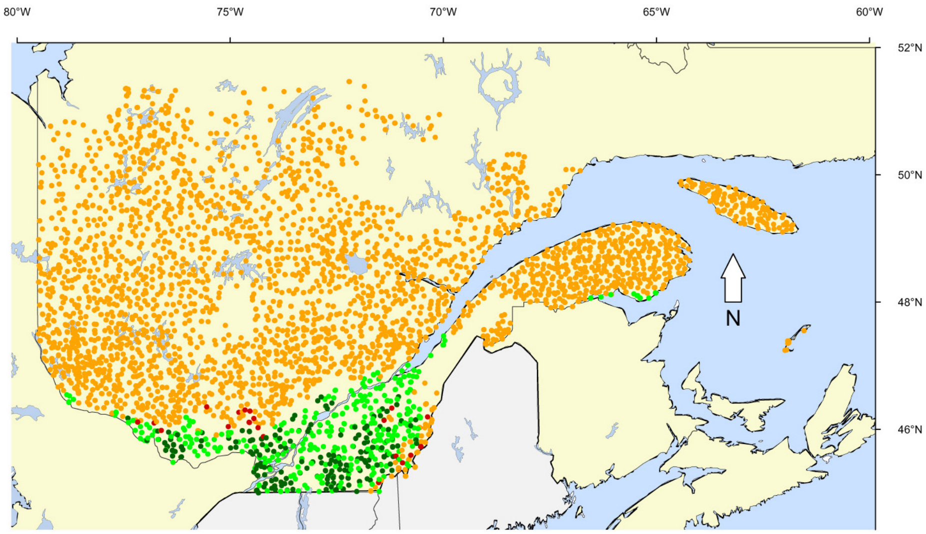

Climatic variables only
Sensitivity: 88.2% - Specificity: 88.2%

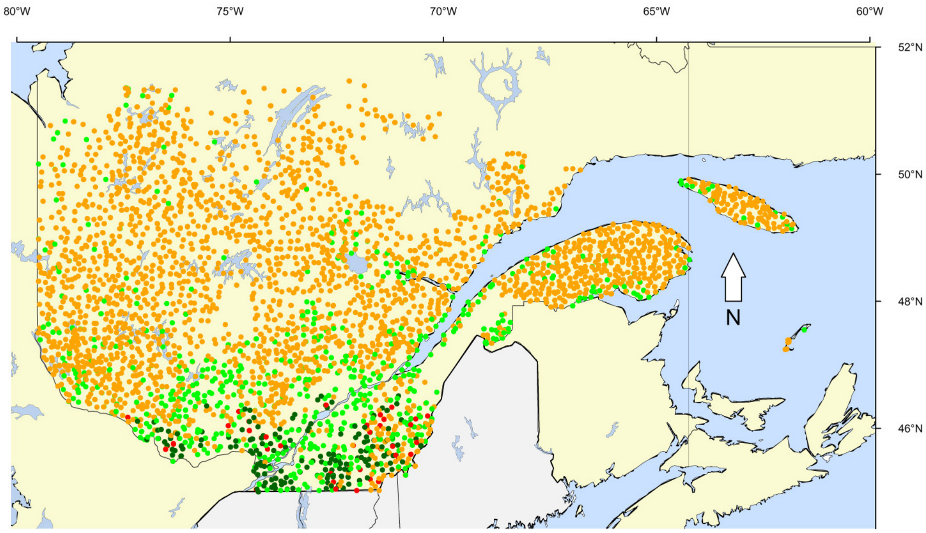

Edaphic variables only
Sensitivity: 86.1% - Specificity: 86.3%

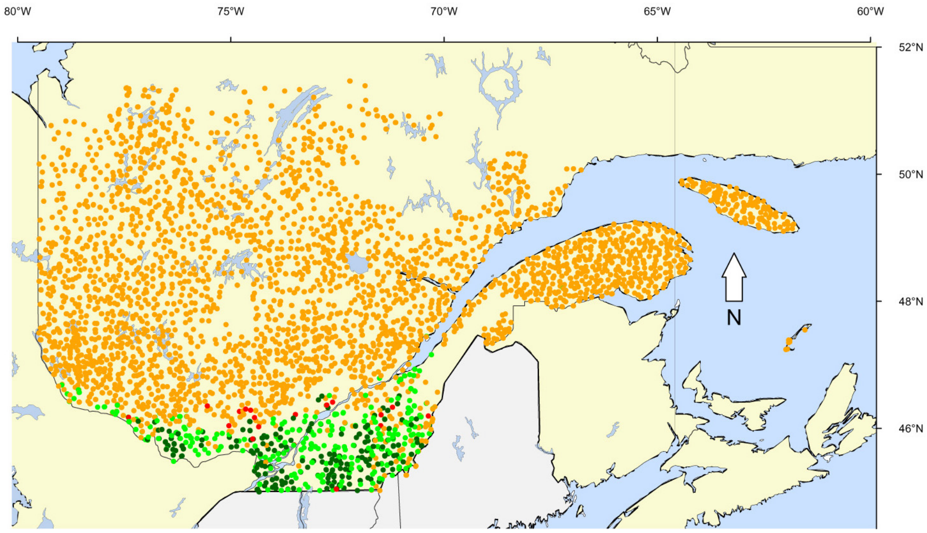

Climatic and edaphic variables
Sensitivity: 90.3% - Specificity: 90.1%
