# Supplementary material for: Beyond a Climate-Centric View of Plant Distribution: Edaphic Variables Add Value to Distribution Models
Source: PLoS One. 2014 Mar 21;9(3):e92642. doi: 10.1371/journal.pone.0092642 (PMC3962442; doi:10.1371/journal.pone.0092642)
Supplement: Table S1 — List of modelled species, their characteristics and model predictive accuracy. Cross-validation scores-are the means of four different statistical model types: generalized boosted models, generalized linear regression models, generalized additive models, and random forest models, each with ten iterations of data-splitting for model building and evaluation.; SDM- species distribution model; AUC-the area under the curve of the receiver operating characteristic; TSS-true skill statistic; Edge- indicates if the species had an observable range boundary within the study points; Count-the count of the number of occurrences. (PDF) [file pone.0092642.s020.pdf]

Table S1. List of modelled species, their characteristics and model predictive accuracy.

				Cross-validation scores					
	Species characteristics			Climate SDM		Edaphic SDM		Edaphic-climate SDM	
Scientific name	Form	Edge	Count	AUC	TSS	AUC	TSS	AUC	TSS
Abies balsamea (L.) Mill.	Tree	-	3966	0.81	0.52	0.74	0.38	0.84	0.54
Acer pensylvanicum L.	Shrub	-	924	0.89	0.66	0.83	0.51	0.91	0.69
Acer rubrum L.	Tree	+	1855	0.89	0.66	0.82	0.51	0.90	0.67
Acer saccharum Marsh.	Tree	+	2290	0.90	0.68	0.87	0.57	0.93	0.72
Acer spicatum Lam.	Shrub	+	296	0.80	0.48	0.80	0.48	0.85	0.56
Actaea rubra (Aiton) Willd.	Herb seed bearing	+	1194	0.74	0.38	0.81	0.49	0.82	0.50
Alnus incana (L.) Moench. ssp. *rugosa* (Du Roi) Clausen	Tree	-	905	0.71	0.34	0.79	0.46	0.82	0.49
Alnus viridis (Chaix) DC. ssp. *crispa* (Aiton) Turrill	Shrub	+	492	0.80	0.49	0.76	0.41	0.82	0.53
Anaphalis margaritacea (L.) Benth.	Herb seed bearing	+	179	0.81	0.54	0.77	0.46	0.82	0.54
Apocynum androsaemifolium L.	Herb seed bearing	+	119	0.71	0.36	0.71	0.35	0.74	0.40
Aralia nudicaulis L.	Shrub	+	2693	0.76	0.42	0.76	0.40	0.81	0.49
Athyrium filix-femina (L.) Roth	Herb seedless	+	784	0.69	0.28	0.76	0.39	0.77	0.42
Bazzania trilobata (L.) Gray	Herb seedless	+	777	0.66	0.25	0.65	0.23	0.70	0.31
Betula alleghaniensis Britton	Tree	+	1266	0.87	0.59	0.82	0.48	0.88	0.61
Betula papyrifera Marsh.	Tree	+	3230	0.73	0.35	0.75	0.39	0.80	0.45
Betula populifolia Marsh.	Tree	-	212	0.95	0.85	0.87	0.63	0.94	0.84
Botrychium virginianum (L.) Swartz	Herb seedless	-	105	0.82	0.53	0.82	0.53	0.85	0.57
Chamerion angustifolium (L.) Holub ssp. *angustifolium*	Herb seed bearing	+	129	0.73	0.36	0.69	0.31	0.75	0.39
Chamaedaphne calyculata(L.) Moench	Shrub	+	630	0.85	0.61	0.85	0.54	0.89	0.68
Chimaphila umbellata (L.) Bartram ssp. *umbellata*	Shrub	+	157	0.88	0.61	0.85	0.56	0.89	0.63
Circaea alpina L.	Herb seed bearing	-	183	0.71	0.35	0.77	0.43	0.78	0.44
Clintonia borealis (Aiton) Raf.	Herb seed bearing	-	877	0.73	0.35	0.76	0.39	0.79	0.45
Cladina mitis (Sandst.) Hustich	Herb seedless	+	1814	0.80	0.49	0.78	0.42	0.83	0.50
Cladina rangiferina (L.) Nyl.	Herb seedless	+	645	0.83	0.52	0.80	0.46	0.86	0.56

Cladina stellaris (Opiz) Brodo	Herb seedless	+	3171	0.89	0.66	0.86	0.59	0.92	0.71
Cornus alternifolia L. f.	Shrub	+	266	0.83	0.53	0.81	0.52	0.85	0.58
Corylus cornuta Marsh.	Shrub	-	357	0.84	0.55	0.80	0.48	0.87	0.60
Cornus stolonifera Michx.	Shrub	-	409	0.77	0.45	0.75	0.41	0.80	0.49
Coptis trifolia (L.) Salisb.	Herb	-	1804	0.61	0.18	0.68	0.27	0.71	0.31
Dennstaedtia punctilobula (Michx.) Moore	Herb seedless	+	2121	0.94	0.81	0.80	0.50	0.95	0.81
Diervilla lonicera Miller	Shrub	-	1390	0.76	0.40	0.75	0.39	0.81	0.48
Dryopteris carthusiana (Vill.) Fuchs	Herb seedless	-	2558	0.75	0.37	0.77	0.41	0.81	0.48
Epigaea repens L.	Herb seed bearing	+	213	0.70	0.32	0.77	0.42	0.79	0.45
Equisetum sylvaticum L.	Herb seedless	+	211	0.84	0.59	0.89	0.67	0.90	0.68
Eurybia macrophylla (L.) Cass.	Herb seed bearing	+	1024	0.79	0.47	0.75	0.39	0.82	0.52
Fagus grandifolia Ehrh.	Tree	+	546	0.93	0.77	0.85	0.58	0.95	0.80
Fraxinus americana L.	Tree	+	243	0.96	0.85	0.88	0.65	0.97	0.85
Fraxinus nigra Marsh.	Tree	+	218	0.82	0.54	0.81	0.50	0.88	0.57
Gaultheria hispidula (L.) Muhl. ex Bigelow	Shrub	-	139	0.81	0.49	0.83	0.50	0.86	0.57
Gaultheria procumbens L.	Shrub	-	1896	0.82	0.50	0.77	0.44	0.86	0.60
Galium triflorum Michx.	Herb seed bearing	+	150	0.77	0.43	0.73	0.38	0.80	0.49
Goodyera repens (L.) Br.	Herb seed bearing	+	170	0.71	0.36	0.69	0.31	0.73	0.39
Gymnocarpium dryopteris (L.) Newmen	Herb seedless	+	843	0.65	0.25	0.69	0.28	0.72	0.33
Huperzia lucidula (Michx.) Trevis.	Herb seedless	+	958	0.71	0.35	0.75	0.37	0.77	0.41
Hylocomium splendens (Hedw.) Schimp.	Herb seedless	-	1240	0.74	0.37	0.73	0.35	0.75	0.40
Ilex mucronata (L.) Powell, Savolainen & Andrews	Shrub	-	983	0.71	0.32	0.74	0.37	0.79	0.45
Impatiens capensis Meerb.	Herb seed bearing	+	132	0.84	0.58	0.78	0.45	0.86	0.60
Kalmia angustifolia L.	Shrub	-	1589	0.84	0.57	0.87	0.59	0.90	0.66
Larix laricina (Du Roi) Koch	Tree	-	222	0.62	0.24	0.75	0.40	0.77	0.42
Ledum groenlandicum Oeder	Shrub	+	1296	0.89	0.65	0.88	0.61	0.92	0.71
Linnaea borealis L.	Shrub	+	1823	0.69	0.29	0.68	0.28	0.73	0.35
Lonicera canadensis Bartram ex Marsh.	Shrub	+	1406	0.82	0.52	0.76	0.41	0.87	0.59
Lycopodium annotinum L.	Herb seedless	+	1053	0.69	0.32	0.67	0.27	0.72	0.36
Lycopodium clavatum L.	Herb seedless	-	548	0.64	0.21	0.65	0.24	0.68	0.28

Lycopodium complanatum L.	Herb seedless	+	157	0.63	0.24	0.67	0.28	0.70	0.32
Lycopodium obscurum L.	Herb seedless	+	1565	0.67	0.26	0.74	0.36	0.76	0.40
Maianthemum canadense Desf.	Herb seed bearing	+	3367	0.65	0.24	0.69	0.29	0.71	0.33
Maianthemum racemosum (L.) Link ssp. *racemosum*	Herb seed bearing	+	419	0.83	0.56	0.81	0.51	0.87	0.62
Maianthemum trifolium (L.) Sloboda	Herb seed bearing	+	222	0.81	0.53	0.88	0.64	0.91	0.71
Medeola virginiana L.	Herb seed bearing	-	314	0.88	0.68	0.81	0.51	0.91	0.70
Mitella nuda L.	Herb seed bearing	+	198	0.79	0.46	0.84	0.55	0.88	0.61
Mitchella repens L.	Shrub	+	448	0.94	0.80	0.85	0.57	0.93	0.78
Moneses uniflora (L.) Gray	Herb seed bearing	+	126	0.77	0.49	0.65	0.28	0.76	0.44
Monotropa uniflora L.	Herb seed bearing	+	334	0.70	0.32	0.66	0.27	0.73	0.36
Oclemena acuminate (Michx.) Greene	Herb seed bearing	+	787	0.75	0.39	0.68	0.30	0.78	0.44
Onoclea sensibilis L.	Herb seedless	+	238	0.90	0.70	0.87	0.62	0.93	0.76
Orthilia secunda (L.) House	Shrub	+	357	0.72	0.39	0.68	0.31	0.73	0.40
Osmunda cinnamomea L.	Herb seedless	+	296	0.80	0.48	0.79	0.47	0.82	0.52
Osmunda claytoniana L.	Herb seedless	+	627	0.72	0.33	0.75	0.38	0.78	0.43
Ostrya virginiana (Mill.) Koch	Tree	+	239	0.94	0.77	0.89	0.67	0.95	0.79
Oxalis montana Raf.	Herb seed bearing	+	1546	0.76	0.39	0.76	0.39	0.81	0.47
Petasites frigidus (L.) Fries var. *palmatus* (Aiton) Cronquist	Herb seed bearing	+	275	0.86	0.60	0.89	0.68	0.91	0.71
Phegopteris connectilis (Michx.) Watt	Herb seedless	+	846	0.72	0.34	0.75	0.39	0.79	0.44
Picea glauca (Moench) Voss	Tree	+	2097	0.85	0.59	0.85	0.54	0.90	0.66
Picea mariana (Mill.) Britton, Sterns & Poggenb.	Tree	-	2539	0.76	0.41	0.72	0.33	0.79	0.46
Picea rubens Sarg.	Tree	-	509	0.89	0.61	0.84	0.53	0.90	0.64
Pinus banksiana Lamb.	Tree	-	765	0.89	0.69	0.89	0.67	0.85	0.75
Pinus strobus L.	Tree	+	424	0.87	0.61	0.74	0.37	0.89	0.64
Pleurozium schreberi (Brid.) Mitt.	Herb seedless	+	3728	0.86	0.60	0.78	0.46	0.88	0.62
Populus balsamifera L.	Tree	+	106	0.85	0.57	0.79	0.47	0.86	0.59
Populus grandidentata Michx.	Tree	+	170	0.75	0.41	0.82	0.54	0.83	0.55
Polygonatum pubescens (Willd.) Pursh	Herb seed bearing	+	323	0.89	0.68	0.81	0.53	0.90	0.70

Populus tremuloides Michx.	Tree	+	1569	0.86	0.60	0.80	0.50	0.89	0.66
Polypodium virginianum L.	Herb seedless	-	104	0.67	0.25	0.69	0.28	0.71	0.32
Prunus pensylvanica L.	Tree	+	1347	0.78	0.47	0.81	0.51	0.86	0.59
Prunus serotina Ehrh.	Tree	-	272	0.66	0.25	0.63	0.20	0.69	0.29
Prunus virginiana L.	Tree	+	388	0.96	0.84	0.87	0.61	0.96	0.85
Pteridium aquilinum var. *latiusculum* (Desv.) Underw. ex Heller	Herb seedless	+	1402	0.86	0.60	0.81	0.49	0.88	0.62
Ptilium crista-castrensis (Hedw.) De Not.	Herb seedless	+	1973	0.74	0.37	0.74	0.37	0.80	0.45
Ptilium ciliare De Not	Herb seedless	+	413	0.81	0.48	0.76	0.40	0.82	0.51
Pyrola elliptica Nutt.	Herb seed bearing	+	139	0.90	0.68	0.84	0.55	0.91	0.69
Quercus rubra L. var. *ambigua* (Gray) Fernald	Tree	+	213	0.70	0.34	0.67	0.29	0.73	0.38
Rhizomnium magnifolium (Horik.) Kop.	Herb seedless	+	104	0.95	0.80	0.83	0.54	0.95	0.80
Rhytidiadelphus triquetrus (Hedw.) Warnst.	Herb seedless	+	307	0.68	0.30	0.67	0.30	0.71	0.36
Ribes glandulosum Grauer	Shrub	+	1114	0.71	0.34	0.70	0.34	0.75	0.39
Ribes lacustre (Pers.) Poir.	Shrub	-	568	0.64	0.20	0.67	0.25	0.70	0.29
Ribes triste Pall.	Shrub	-	255	0.78	0.49	0.80	0.49	0.82	0.53
Rubus idaeus L.	Shrub	-	1459	0.66	0.25	0.70	0.30	0.73	0.34
Rubus pubescens Ruf.	Shrub	+	1248	0.69	0.28	0.78	0.42	0.78	0.43
Sambucus racemosa L.	Shrub	-	945	0.75	0.38	0.74	0.37	0.78	0.43
Sorbus americana Marsh.	Tree	-	1673	0.68	0.27	0.68	0.28	0.73	0.36
Sorbus decora (Sarg.) Schneid.	Tree	-	710	0.82	0.51	0.79	0.45	0.84	0.54
Solidago macrophylla Pursh.	Herb seed bearing	+	606	0.79	0.44	0.75	0.39	0.80	0.47
Solidago rugosa Mill.	Herb seed bearing	+	124	0.86	0.57	0.80	0.50	0.88	0.66
Spiraea alba du Roi	Shrub	+	151	0.89	0.66	0.85	0.61	0.91	0.71
Sphagnum fuscum (Schimp.) Klinggr.	Herb seedless	+	433	0.82	0.51	0.81	0.50	0.87	0.62
Sphagnum girgensohnii Russow	Herb seedless	+	580	0.70	0.31	0.78	0.44	0.80	0.47
Sphagnum magellanicum Brid.	Herb seedless	+	221	0.69	0.31	0.85	0.58	0.85	0.58
Sphagnum squarrosum Crome	Herb seedless	+	123	0.58	0.17	0.75	0.43	0.74	0.42
Streptopus amplexifolius (L.) DC.	Herb seed bearing	+	237	0.68	0.31	0.64	0.24	0.68	0.30
Streptopus lanceolatus (Aiton) Reveal var.*lanceolatus*	Herb seed bearing	+	1338	0.70	0.31	0.73	0.36	0.76	0.40

Taxus canadensis Marsh.	Shrub	+	517	0.74	0.37	0.72	0.34	0.77	0.41
Thelypteris noveboracensis (L.) Nieuwl.	Herb seedless	+	426	0.83	0.54	0.78	0.44	0.85	0.58
Thuja occidentalis L.	Tree	+	672	0.82	0.54	0.74	0.38	0.85	0.57
Thalictrum pubescens Pursh	Herb seed bearing	+	116	0.66	0.26	0.71	0.34	0.75	0.41
Tilia americana L.	Tree	+	212	0.95	0.81	0.91	0.69	0.96	0.82
Tiarella cordifolia L.	Herb seed bearing	+	267	0.92	0.74	0.85	0.57	0.93	0.75
Trientalis borealis Raf.	Herb seed bearing	+	2798	0.69	0.29	0.70	0.30	0.74	0.36
Trillium erectum L.	Herb seed bearing	+	618	0.85	0.57	0.82	0.51	0.87	0.60
Trillium undulatum Willd.	Herb seed bearing	+	414	0.82	0.51	0.74	0.37	0.84	0.52
Tsuga canadensis (L.) Carriere	Tree	+	244	0.94	0.81	0.87	0.61	0.95	0.82
Ulmus americana L.	Tree	+	153	0.95	0.79	0.92	0.70	0.94	0.78
Vaccinium angustifolium Aiton	Shrub	-	1841	0.82	0.52	0.80	0.49	0.85	0.56
Vaccinium myrtilloides Michx.	Shrub	-	2183	0.79	0.46	0.80	0.45	0.83	0.51
Vaccinium oxycoccos L.	Shrub	-	111	0.92	0.73	0.92	0.74	0.95	0.82
Viburnum edule (Michx.) Raf.	Shrub	-	423	0.77	0.44	0.75	0.38	0.81	0.49
Viburnum lantanoides Michx.	Shrub	+	675	0.88	0.65	0.86	0.55	0.91	0.68
Viburnum nudum L. var. *cassinoides* (L.) Torr. & A. Gray	Shrub	+	1417	0.81	0.49	0.77	0.40	0.85	0.55

Cross-validation scores-are the means of four different statistical model types: generalized boosted models, generalized linear regression models, generalized additive models, and random forest models, each with ten iterations of data-splitting for model building and evaluation.; *SDM*- species distribution model; *AUC*-the area under the curve of the receiver operating characteristic; *TSS*-true skill statistic ; *Edge*- indicates if the species had an observable range boundary within the study points; *Count*-the count of the number of occurrences.
