# Supplementary material for: Beyond a Climate-Centric View of Plant Distribution: Edaphic Variables Add Value to Distribution Models
Source: PLoS One. 2014 Mar 21;9(3):e92642. doi: 10.1371/journal.pone.0092642 (PMC3962442; doi:10.1371/journal.pone.0092642)
Supplement: Table S2 — List of climate variables included in the VARCLUS analysis. (PDF) [file pone.0092642.s021.pdf]

Table S2. List of climate variables included in the VARCLUS analysis.

Average annual temperature (°C)

Annual temperature range (coldest month, warmest month) (C°)

Growing degree days (5°C base temperature)

Growing degree days accumulated within the frost free period

Julian date of the last spring freeze

Julian date of the first fall freeze

Length of frost free period (days)

Julian date when the sum of growing degree days reaches 100

Total annual precipitation (mm)

Total precipitation from April to September (mm)

Total precipitation of the wettest month (mm)

Total precipitation of the driest month (mm)
