# Supplementary material for: Beyond a Climate-Centric View of Plant Distribution: Edaphic Variables Add Value to Distribution Models
Source: PLoS One. 2014 Mar 21;9(3):e92642. doi: 10.1371/journal.pone.0092642 (PMC3962442; doi:10.1371/journal.pone.0092642)
Supplement: Table S3 — Model predictive accuracy and the influence of species biogeographic characteristics on these metrics. AUC-the area under the curve of the receiver operating characteristic; TSS-true skill statistic; SDM-species distribution model. Reported are the means of the final climate-edaphic model for each statistical model type; comparisons of the presence of a range boundary in the study area (t-test) and the effect of number of occurrences (Pearson correlation) significant results are in bold, *P<0.1; **P<0.05. (PDF) [file pone.0092642.s022.pdf]

Table S3. Model predictive accuracy and the influence of species biogeographic characteristics on these metrics.

	**Range boundary** (mean) n = 94	**No range boundary** (mean) n = 34	**Prevalence** R n = 128
**Climate SDM**			
AUC	**0.87**	**0.83****	**-0.25****
TSS	**0.64**	**0.57***	**-0.29****
**Edaphic SDM**			
AUC	**0.86**	**0.84****	**-0.39****
TSS	**0.63**	**0.58****	**-0.41****
**Edaphic-climate SDM**			
AUC	0.89	0.89	-0.04
TSS	**0.69**	**0.64***	-0.09

AUC-the area under the curve of the receiver operating characteristic; *TSS*-true skill statistic; *SDM*-species distribution model. Reported are the means of the final climate-edaphic model for each statistical model type; comparisons of the presence of a range boundary in the study area (t-test) and the effect of number of occurrences (Pearson correlation) significant results are in bold, *P < 0.1; **P < 0.05.
