# Supplementary material for: Beyond a Climate-Centric View of Plant Distribution: Edaphic Variables Add Value to Distribution Models
Source: PLoS One. 2014 Mar 21;9(3):e92642. doi: 10.1371/journal.pone.0092642 (PMC3962442; doi:10.1371/journal.pone.0092642)
Supplement: Table S4 — Differing importance of variables between plant growth form groups. Reported are significant ANOVA results of variable importance (calculated as one minus the correlation between the model output and the model output with the variable of interest randomized) for the means of the final climate-edaphic model for each statistical model type between the plant form groups: tree, shrub, herbaceous seed bearing, and seedless plants (including lichens). (PDF) [file pone.0092642.s023.pdf]

Table S4. Differing importance of variables between plant form groups.

	Degree days	Soil depth	Texture
MS	0.451	0.001	0.007
F	8.684	4.666	2.811
P	0.001	0.004	0.042
Means			
Trees (n = 30)	0.501	0.007	0.029
Shrubs (n = 34)	0.271	0.008	0.057
Herbaceous (n = 32)	0.279	0.019	0.057
Seedless (n = 32)	0.233	0.017	0.034
Tukey’s HSD results	trees>all others	herbaceous>trees, shrubs	none
LSD test	trees>all others	herbaceous>trees, shrubs	Herbaceous, shrubs>trees

Reported are significant ANOVA results of variable importance (calculated as one minus the correlation between the model output and the model output with the variable of interest randomized) for the means of the final climate-edaphic model for each statistical model type between the plant form groups: tree, shrub, herbaceous seed bearing, and seedless plants (including lichens)
